# Noise2Kernel: Adaptive Self-Supervised Blind Denoising Using a Dilated Convolutional Kernel Architecture

**DOI:** 10.3390/s22114255

**Published:** 2022-06-02

**Authors:** Kanggeun Lee, Won-Ki Jeong

**Affiliations:** Department of Computer Science and Engineering, Korea University, Seoul 02841, Korea; leekanggeun@korea.ac.kr

**Keywords:** blind denoising, self-supervision, adaptive loss, J-invariant network

## Abstract

With the advent of unsupervised learning, efficient training of a deep network for image denoising without pairs of noisy and clean images has become feasible. Most current unsupervised denoising methods are built on self-supervised loss with the assumption of zero-mean noise under the signal-independent condition, which causes brightness-shifting artifacts on unconventional noise statistics (i.e., different from commonly used noise models). Moreover, most blind denoising methods require a random masking scheme for training to ensure the invariance of the denoising process. In this study, we propose a dilated convolutional network that satisfies an invariant property, allowing efficient kernel-based training without random masking. We also propose an adaptive self-supervision loss to increase the tolerance for unconventional noise, which is specifically effective in removing salt-and-pepper or hybrid noise where prior knowledge of noise statistics is not readily available. We demonstrate the efficacy of the proposed method by comparing it with state-of-the-art denoising methods using various examples.

## 1. Introduction

Denoising is one of the actively studied topics in computer vision and image processing. Images generated from various devices are prone to noise and corruption due to limited imaging environments (e.g., low light, slow shutter speed, etc.). Conventional denoising methods usually rely on known noise models based on specific noise distributions. For instance, image prior-based approaches, such as self-similarity [1,2,3,4,5], require a specific property of pre-defined noise statistics or prior knowledge of a target image. However, there exist many real examples that pre-defined noise statistics do not fit, such as the coherent random noise observed in the transparent films used in electron microscopy (EM) imaging [6]. In such cases, conventional denoising methods may not work well.

In recent years, the supervised learning of convolutional neural networks (CNNs) which have been widely used in many tasks [7,8,9] using clean-noisy image pairs has achieved superior denoising performance [10,11]. Due to the difficulty in obtaining clean–noisy image pairs in real examples, Lehtinen et al. [12], in their seminal study, introduced unsupervised learning of a denoiser (Noise2Noise (N2N)) using only noisy images. Even though N2N proposed the general approach, it still suffers from the acquisition of noisy-noisy image pairs under known noise statistics.

**Figure 1:**
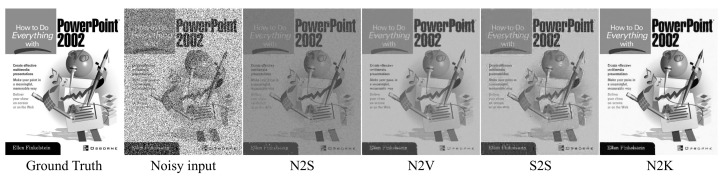
Denoising results on the image highly corrupted by non-zero mean salt-and-pepper noise. Note that the results of N2S, N2V, and S2S look much darker than the ground truth. Our proposed method (N2K) successfully removes noise without brightness shifting.

More recently, several new unsupervised image denoising methods [13,14,15,16] have shown promising results with denoisers that can be trained in a self-supervised fashion. For example, Noise2Void (N2V) [14] and Noise2Self (N2S) [15] require only the assumption of zero-mean noise without prior knowledge of noise statistics. These methods performed the denoising task successfully using only noisy images under a zero-mean noise condition. Self2Self (S2S) [16] proposed a novel framework with dropout using Bernoulli-sampled instances of a single input image. Moreover, using only a single training image, S2S outperformed on several famous noise distributions. Despite their potential, these approaches have several drawbacks. First, these self-supervised methods approximate the optimal denoiser with a noisy distribution based on the blind-spot scheme (i.e., random masking of pixels during training). The blind-spot scheme damages the original noisy image, and the large masking rate leads to poor performance. Second is the weakness of the general self-supervision loss function. Because general self-supervision training depends on only the noisy signal, excessive noise causes CNNs to learn poorly and incorrectly. We discovered that state-of-the-art blind denoising methods are prone to predicting the wrong brightness level or shape if noisy images are corrupted by impulse noise (e.g., salt-and-pepper noise) or unconventional noise (e.g., fusion noise). Even though S2S successfully removed the pepper noise, it also predicted different brightness for salt-and-pepper noise, as shown in Figure 1. As a result, for state-of-the-art blind denoising methods, the brightness-shifting artifact always appears in the case of corrupted salt-and-pepper noise.

To address the above issues, we introduce a novel unsupervised-learning-based denoising network. The combination of dilated convolution layers and donut-shaped kernels means it can build a specific function that satisfies the J-invariant property [15]. In addition to the novel network architecture, we propose a novel adaptive self-supervision (ADSS) loss to restore the clean signal on the highly corrupted noisy image without brightness shifting.

The main contributions of our study are as follows:

1. We propose a dilated convolutional invariant network using a donut-shaped kernel and dilated convolutional layers. We no longer need a special training scheme (e.g., random masking) for blind denoising with self-supervision loss.
2. We propose an adaptive self-supervision loss, which is the pixel-level nonlinear energy, to suppress incorrect learning from unconventional noise. We demonstrate that the proposed adaptive loss is highly effective on corrupted noisy images (for example, images with speckle noise, salt-and-pepper noise, and fusion noise) without any prior knowledge of the noise model.
3. We demonstrate that the total variation regularization term can help to restore the pixel-wise artifact, which is a drawback of the proposed method.

To the best of our knowledge, the proposed method is the first fully blind denoising method that can prevent brightness shifting for images highly corrupted by speckle noise, salt-and-pepper noise, and fusion noise without noise statistics and clean–noisy pairs.

## 2. Related Work

### 2.1. Conventional Denoising Methods

Total variation (TV), known as TV regularization, is a widely used denoising technique [17,18,19,20] that adopts prior sparsity gradients in image denoising. Filtering methods [1,2,21] based on spatial information or nonlocal self-similarity achieve better performance than TV-based methods. The block-matching and 3D filtering (BM3D) algorithm [2] still performs well enough to be used as a comparison for deep learning. The structure of BM3D is actively applied to various noise types, including salt-and-pepper noise and speckle noise [22,23]. With efficient training data, learning-based methods eventually perform better than non-learning-based ones. Before deep learning, which involves the use of large training data, dictionary learning (DL) and convolutional sparse coding (CSC) [24] were used to restore the original signal using a sparse representation prior with a learned dictionary [25,26,27,28].

### 2.2. Non-Blind Denoising Methods

In recent years, with advances in deep learning and the related equipment, supervised deep learning over CNNs [10,29,30] has shown great promise with its denoising performance. However, it is not suitable to apply this method in practice because most supervised learning methods require noise statistics to generate the training data on a clean dataset.

Recently, Noise2Noise (N2N) [12] proved that training a deep learning model is feasible and that the expected value of noisy inputs could be equal to the clean target. However, in situations where the noise statistics are unknown, N2N is impractical because of the difficulty in collecting a noisy pair for the same target. With only noise statistics, these works [31,32] perform as well as or slightly better than supervised learning. For instance, Ref. [31] suggest concrete self-supervision losses suitable for each noise statistic, but it is difficult to apply the proposed loss in cases with unknown noise statistics. Ref. [31] also presented a new blind-spot network that contributes similar to the architecture presented in the present study. However, we take a different approach to enabling self-supervision learning using the J-invariant property. Similarly, Noisy-As-Clean (NAC) [32] suggested a training scheme with pairs of noisy images *x* and $x+n_{s}$ where $n_{s}$ is a simulated noise. The researchers demonstrated that loss function $L(f\left(x+n_{s}\right),x)$ can be embedded into supervised learning. Noisier2Noise [33] presented a novel training approach with only a single noisy realization and noise statistics. It also overcomes the drawback of N2N that is the requirement of a prior of noise distribution. Moreover, the Noisier2Noise approach is applicable to spatially structured noise, one of the main disadvantages of a blind denoising method.

### 2.3. Blind Denoising Methods

Blind denoising approaches assume that the prior noise distribution is unknown. To restore the clean signal without noise statistics, for instance, deep image prior (DIP) [13] tries to use a handcrafted prior to the image processing tasks. In other words, DIP shows that image prior can be learned by a random-initialized neural network without a specific condition. However, the internal-image-prior-based approach has the two drawbacks of excessive testing time and inadequate performance.

The external-image-prior-based approaches, such as N2V [14] and N2S [15], employ the blind-spot scheme to prevent being an identity mapping function by self-supervision loss. Furthermore, two state-of-the-art methods take the self-supervision perspective to train the deep learning model using only noisy images. Two methods achieved significant shortening of testing time through the external image prior. In addition, N2S [15] suggested the J-invariant property to prove that self-supervision loss can substitute for general loss of supervised learning.

Recently, S2S [16] proposed a novel framework based on Bernoulli dropout, a new masking scheme in the training step, to avoid increasing the variance based on internal image prior because a single training sample causes large variances for denoising models such as a Bayes estimator. Even though only a single noisy image is a training sample, S2S outperforms existing blind denoising methods based on the external image prior.

## 3. Method

**Figure 2:**
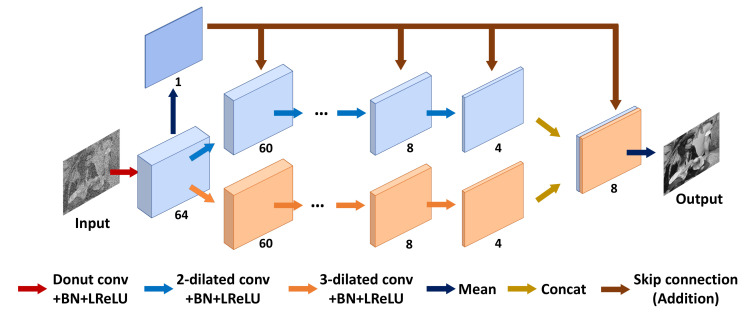
Overview of the network structure of Noise2Kernel.

In this section, we introduce a novel deep neural network architecture that satisfies the J-invariant property for blind noise reduction using adaptive self-supervision learning (Figure 2). First, we reiterate the definition of J-invariant, originally introduced in N2S [15]. Next, we demonstrate that the proposed network satisfies the J-invariant, which allows self-supervised training without using a specific training scheme (e.g., random masking). Finally, we suggest the adaptive self-supervision loss to overcome the drawback of the conventional self-supervision loss.

### 3.1. Formulations

This section introduces the formal definition and proposition of J-invariant that is required to explain the proposed network (more details can be found in N2S).

**Definition** **1.** *Let J be a partition of the dimensions {1,…,m}. Let x be an observed noisy signal, and $x_{J}$ be a sub-sample of x restricted to J∈J. A function $f:R^{m}\to R^{m}$ is J-invariant if the value of $f{\left(x\right)}_{J}$ does not depend on the value of $x_{J}$; f is J-invariant if it is J-invariant for each J∈J.*

We employ self-supervision loss as follows to restore the noisy image using the J-invariant function *f*.

(1) $$\begin{array}{c} L\left(f,x\right)=||f\left(x\right)-{x||}^{2} \end{array}$$

To demonstrate that self-supervision loss can take the place of supervised loss, we borrow the same proposition from N2S under the J-invariant definition.

**Proposition** **1.** *Let us assume that observed image x is an unbiased estimator of y. Let f be the J-invariant function. Then*

(2) $$\begin{array}{c} E||f\left(x\right)-{x||}^{2}=E||f\left(x\right)-{y||}^{2}+E||x-{y||}^{2} \end{array}$$

**Proof.**  Let us consider the self-supervision loss over *f* function.

(3) $$\begin{array}{c} E_{x}||f\left(x\right)-{x||}^{2}=E_{x,y}||f\left(x\right)-y-{\left(x-y\right)||}^{2}\\ =E_{x,y}||f\left(x\right)-{y||}^{2}+||x-{y||}^{2}-2\langle f\left(x\right)-y,x-y\rangle \end{array}$$

The inner product term 〈f(x)−y,x−y〉 can be considered as follows:

(4) $$\begin{array}{c} \Sigma_{i}E_{y}\left(E_{x|y}\left[\left(f{\left(x\right)}_{i}-y_{i}\right)\left(x_{i}-y_{i}\right)\right]\right) \end{array}$$

Because $f{\left(x\right)}_{i}|y$ and $x_{i}|y$ are independent due to the invariant property of *f*, Equation (4) becomes $\Sigma_{i}E_{y}\left(E_{x|y}\left[f{\left(x\right)}_{i}-y_{i}\right]\right)\left(E_{x|y}\left[x_{i}-y_{i}\right]\right)$. Then, the third term of Equation (3) vanishes since $E_{x|y}\left[x_{i}-y_{i}\right]$ is zero due to the zero-mean assumption of noise. □

From this, we can infer that the general self-supervised loss would be the sum of the general supervised loss and the variance of noise. Therefore, based on the similar scheme of N2S, we can conclude that an invariant function *f* can be a general denoiser if *f* is minimized using a self-supervision loss. In the following section, we introduce the proposed network, which is an J-invariant function using a donut-shaped kernel-based convolution layer and dilated convolutional layers.

### 3.2. Dilated Convolutional J-Invariant Network

**Figure 3:**
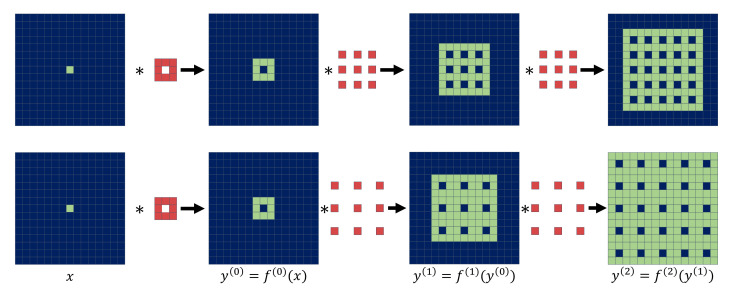
An example of dependency between the pixels in the input (*x*) and the output (*y*) images with one 3×3 donut convolution filter and *d*-dilated convolution. Each row represents the dependency visualization with two 2-dilated convolution and two 3-dilated convolution layers, respectively. The green pixels indicate the pixel locations that have dependency with $x_{i,j}$ (the center pixel in *x*). The red pixels represent the trainable variables of the convolution kernels. The blue pixels indicate the area independent of $x_{i,j}$. This figure shows the intermediate convolution processes of $y=f\left(x\right)=f^{\left(2\right)}\left(f^{\left(1\right)}\left(f^{\left(0\right)}\left(x\right)\right)\right)$ from the input image to the output prediction.

Assume that the function *f* is a CNN with a single donut-shaped kernel (center weight is always zero) (see Figure 3). Based on Definition 1, function *f* satisfies the J-invariant property because $x_{i}$ is the sum of the multiplication of the neighboring information with the donut kernel, except $x_{J}$, for all J∈J where the size of the squared donut kernel *K* is always an odd number. We focus on this J-invariant function in a fully convolutional network (FCN). If only one general convolution layer is added, the invariant property is not satisfied even though the first layer may use the donut kernel. Furthermore, the receptive field of a single layer is too small to predict the correct pixel within the kernel.

Let *f* be a network as a function that consists of *d*-dilated convolution $f^{\left(k\right)}$ ([34]) for all k∈[1,n] where the size of the kernel is 3×3. We infer the function *f* as $f\left(x\right)=f^{\left(n\right)}\left(f^{\left(n-1\right)}\left(\ldots f^{\left(1\right)}\left(f^{\left(0\right)}\left(x\right)\right)\right)\right)$ where $f^{\left(0\right)}$ and *x* are a convolution layer of the K×K donut-shaped kernel and an input noisy image, respectively, and $y^{\left(k\right)}$ is the output features for each *k*-th convolution layer. We then need to demonstrate that $f{\left(x\right)}_{J}$ does not depend on $x_{J}$ for all J∈J.

**Proposition** **2.** *The proposed network f is J-invariant if d≥[K/2].*

**Proof.**  Without loss of generality, we consider a one-dimensional case to prove this proposition. Let us choose one pixel $x_{J}$ where J∈J. Because of the donut convolution layer $f^{\left(0\right)}$, $x_{J}$ information moves to the neighboring region {J−[K/2],…,J−1,J+1,…,J+[K/2]} as shown in Figure 3. Let us suppose that the receptive field of $x_{J}$ in $y^{\left(k\right)}$ is $RF(y^{\left(k\right)},x_{J})$. Then,

(5) $$\begin{array}{c} RF\left(y^{\left(k\right)},x_{J}\right)=\underset{j\in \{-d,0,d\}}{⋃}\left\{i+j|i\in RF\left(y^{\left(k-1\right)},x_{J}\right)\right\} \end{array}$$

where $RF\left(y^{\left(0\right)},x_{J}\right)=\left\{J-\left[K/2\right],\ldots ,J-1,J+1,\ldots ,J+\left[K/2\right]\right\}$ for all k∈[1,n]. By this recurrence relationship, we can infer that [K/2]−d<0 and −[K/2]+d>0 lead to exclude an element *J* in $RF(y^{\left(n\right)},x_{J})$. In other words, the $f{\left(x\right)}_{J}$ never consists of the information of $x_{J}$ if d≥[K/2]. □

The combined structure of the donut convolution layer and dilated convolution layer, as shown in Figure 2, always guarantees the existence of the J-invariant property if d≥[K/2] and the size of the square kernel of donut convolution layer *K* is an odd number. In addition, as shown in Figure 2, there are two paths that both consist of 2-dilated or 3-dilated convolutional layers only. Because each path satisfies the J-invariant condition, the proposed network is J-invariant. According to Equation (5), the combination of two receptive fields of 2-dilation and 3-dilation paths can supplement each other the missing regions (i.e., blue pixels in Figure 3) of a 2-dilated convolutional architecture while increasing the field-of-view. To preserve the first prediction computed by a donut kernel, we added a skip connection after the dilated convolution operation. We discovered that the skip improved the convergence speed and image quality. In addition to its model architecture, another important benefit of the proposed method is that it no longer requires the masking scheme. A masked input $\tilde{x}$ of the noisy image *x* with the dimension J⊂J (chosen randomly) is defined as

(6) $$\begin{array}{c} \tilde{x}=\left\{\begin{array}{ccc} 0 & \mathrm{for} & j\in J\\ x_{j} & \mathrm{for} & j\notin J \end{array}\right. \end{array}$$

Then, the general self-supervision loss with the masking scheme is defined as follows:

(7) $$\begin{array}{c} \min_{\theta }\sum_{i}^{N}\sum_{J\subset J}\left|\right|\left(f{\left({\tilde{x}}^{i};\theta \right)}_{J}-x_{J}^{i}\right){\left|\right|}^{2} \end{array}$$

Because random pixel discarding in the masking scheme introduces defects in image ($\tilde{x}$), N2V and N2S fill in these missing pixels by copying from random locations or through interpolation from neighboring pixels. Unlike such existing methods, the dilation convolution architecture of the proposed network can be trained using only the original *x* and the general self-supervision loss without a masking scheme shown below:

(8) $$\begin{array}{c} L\left(f,x\right)=||f\left(x\right)-{x||}^{2} \end{array}$$

### 3.3. Adaptive Self-Supervision Loss

In the unsupervised denoising problem, the zero-mean noise is considered a default noise model. However, the zero-mean condition is too strict to be used on blind denoising with self-supervision loss. For example, in the case of salt noise (i.e., random white dots), the general self-supervision loss may falsely treat the correct prediction as a noisy label due to large differences between the predicted and noisy pixel values, which causes brightness shifting toward white. This implies that self-supervision may fail to work on highly corrupted impulse noise. An additional constraint is required to avoid the convergence to the biased estimator. To address such limitations of standard self-supervision loss, we propose ADSS loss using the focusing parameter λ as follows:

(9) $$\begin{array}{c} L_{adap}\left(f,x\right)=E_{j}\left[w_{j}{\left(f{\left(x\right)}_{j}-x_{j}\right)}^{2}\right] \end{array}$$

(10) $$\begin{array}{c} w_{j}=\frac{1}{{1+\lambda |f\left(x\right)}_{j}-x_{j}|} \end{array}$$

where $x_{j}$ is a pixel indicated by an index *j*. The ADSS loss adjusts the proportion of difference between *x* and f(x) adaptively. The main idea behind ADSS is that, if the prediction is significantly different from the input pixel value, it is highly likely that the input pixel is noise. Therefore, during the training process, backpropagation from such pixels should be suppressed (i.e., the correct predictions should not be shifted toward the noise pixel values) by adaptive control of the weight in the loss function. The ADSS loss is equivalent to the self-supervision loss when λ=0. Intuitively, λ controls the extent of the influence of discrimination. We expect the ADSS loss can avoid unnecessary learning from unpredictable noise.

## 4. Results

To assess the performance of the proposed method, we tested it on various noise models, such as those with additive white Gaussian noise (AWGN), speckle noise, and salt-and-pepper noise. In particular, because we focus on highly corrupted noisy images by unconventional noise in the blind aspect, the noise should be modeled by unknown distribution. To simulate this, we built a fusion noise model by mixing AWGN, speckle noise, and salt-and-pepper noise. We compared our method with several state-of-the-art blind denoising methods (N2V, N2S, and S2S). In addition, we also compared our proposed method with conventional denoising methods, such as BM3D [2], SAR-BM3D [23], and AT2F [35], known for the best performing filter-based denoising method specifically designed for each noise model. We implemented Noise2Clean (N2C) on using the same network structure as shown in Figure 2, with a regular 3×3 convolution kernel for supervised training using the clean–noisy pairs introduced in Section 4.2. Please note that N2C is a supervised learning method, which serves as the upper bound for the performance of the learning-based denoising method.

For all training (except for N2C), we used only noisy images corrupted by simulated noise. We chose the same dataset, BSD400, of gray scale images used in [10,14] as a training dataset. For more detail, we applied augmentation using rotation and mirroring for all learning-based methods. For testing the performance, we employed the BSD68 and Set14 datasets. In particular, S2S experiments on BSD68 were excluded because S2S is inner-prior-based denoising approach that causes the large computational cost. We used the BSD68 dataset for the ablation study as a validation set.

We used TensorFlow [36] (version 2.0.0) to implement the proposed architecture, as shown in Figure 2. For stable performance, we applied an exponential learning rate decay with an RAdam [37] optimizer. We used batch size 64 and 0.03 as the initial learning rate and λ=10 for Equation (9). For a fair comparison, we used the default parameter settings from the authors’ code for other blind denoising methods. We picked the best hyperparameters for experimental comparison methods when the setting of a hyperparameter was required. Because the denoiser should satisfy rotation invariance, we rotated each test image by 90 degrees and made two mirrored versions. The average of the inverse of eight outputs was the final prediction.

To evaluate the image quality, we employ two full-reference image quality assessment (FR-IQA) metrics such as peak signal-to-noise ratio (PSNR) and structural similarity index metric (SSIM) defined as follows:

(11) $$\begin{array}{c} PSNR\left(\hat{y},y\right)=20log_{10}\left(\frac{MAX_{y}}{\sqrt{MSE(\hat{y},y)}}\right), \end{array}$$

where $MAX_{x}$ is the maximum possible pixel value of the image, MSE is the mean squared error. y and $\hat{y}$ are the ground-truth and restored image, respectively. SSIM is defined as follows:

(12) $$\begin{array}{c} SSIM\left(\hat{y},y\right)=\frac{\left(2\mu_{y}\mu_{\hat{y}}+c_{1}\right)\left(2\sigma_{y,\hat{y}}+c_{2}\right)}{\left(\mu_{y}^{2}+\mu_{\hat{y}}^{2}+c1\right)\left(\sigma_{y}^{2}+\sigma_{\hat{y}}^{2}+c_{2}\right)}, \end{array}$$

where $\mu_{y},\mu_{\hat{y}}$ are the average of each image, $\sigma_{y,\hat{y}}$ is the covariance of *y* and $\hat{y}$, and $\sigma_{y},\sigma_{\hat{y}}$ are the variance of each image. For the constants $c1={\left(k_{1}L\right)}^{2}$ and $c2={\left(k_{2}L\right)}^{2}$, we set to $k_{1}=0.01$ and $k_{2}=0.03$. *L* is the dynamic range of pixel values.

### 4.1. Denoising Results on Known Noise Models

#### 4.1.1. Additive White Gaussian Noise (AWGN)

**Figure 4:**
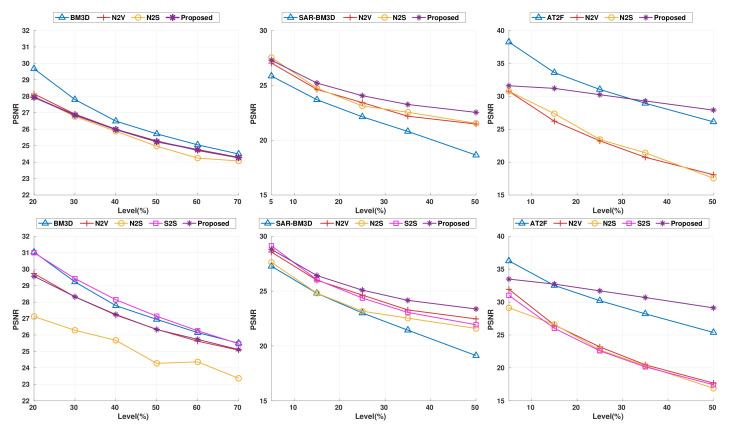
Quantitative performance comparison of various denoising methods on known noise models in the BSD68 and Set14 dataset. Left to right: AWGN, speckle noise, and salt-and-pepper, respectively. Top to bottom: BSD68 and Set14.

AWGN is a popular statistical noise model with a zero-mean characteristic as follows:

(13) $$\begin{array}{c} y=x+n,\;n∼N(0,\;\sigma_{g}^{2})\; \end{array}$$

where N is a normal distribution with standard deviation $\sigma_{g}$. For the baseline performance, we chose BM3D, which is known for being the best performing method for this noise model. For a fair comparison, we used the standard deviation $\sigma_{g}$ (i.e., noise level) of the given noise-corrupted images only for the case of BM3D (without the noise level prior, BM3D does not produce correct results). Figure 4 shows the quantitative performance comparison of denoisers over various noise levels, $\sigma_{g}$ from 20 to 70. The proposed method achieves similar or better performance than N2V and N2S, whereas S2S and BM3D outperformed the proposed method. We expect that the proposed method has no significant performance improvement compared with blind denoising methods on additive white Gaussian noise distribution that satisfy the zero-mean condition. Therefore, we conclude that under the zero-mean noise constraint, our method is comparable to most of the blind denoising methods except S2S.

#### 4.1.2. Speckle Noise

**Figure 5:**
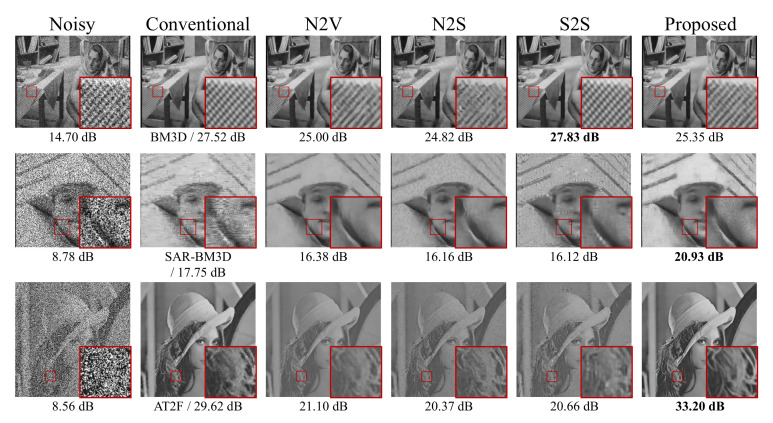
Qualitative performance comparison of various denoising methods on three noise types. Top to bottom row: AWGN ($\sigma_{g}$ = 50), speckle noise ($\sigma_{s}$ = 50), and salt-and-pepper noise (*d* = 50), respectively. The best PSNR in each case is highlighted in bold. Each row indicates the results of AWGN, speckle noise, and salt-and-pepper noise, respectively.

Signal-dependent multiplicative speckle noise, often observed in synthetic aperture radar and ultrasound images, can be modeled as follows:

(14) $$\begin{array}{c} y=x+n*x,\;n∼U(0,\;\sigma_{s}^{2})\; \end{array}$$

where U is the uniform distribution with a zero mean and a standard deviation of $\sigma_{s}$. We chose SAR-BM3D [23], one of the conventional denoising methods specifically designed for speckle noise, as the baseline method to compare with our proposed method. We conducted the denoising experiment over various noise levels $\sigma_{s}$ from 5 to 50. Interestingly, blind denoising methods outperformed SAR-BM3D, as shown in the second column of Figure 4. Please note that the proposed method consistently outperformed the other blind denoising methods for all noise levels $\sigma_{s}$ we tested (see the middle graph in Figure 4). Furthermore, the performance gap between blind denoising methods and SAR-BM3D increases as the noise level increases, which implies that blind denoising methods are more robust to strong speckle noise than SAR-BM3D. The proposed approach achieved the best difference compared to other blind denoisers (by around 4.55 dB higher) on the foreman image in the Set14 dataset (the second row of Figure 5). Moreover, the overall intensity distribution in the predicted image of the proposed method is closer to that of the ground truth; those of other blind denoisers (N2V, N2S, and S2S) suffer from brightness shifting due to the non-zero-mean noise characteristic.

#### 4.1.3. Salt-and-Pepper Noise

In this experiment, we employed salt-and-pepper noise, defined as follows:

(15) $$\begin{array}{c} y=f_{spn}\left(x,d\right) \end{array}$$

where $f_{spn}$ is the projection function set to 0 or 1 with probability *d*. Conventional nonlinear denoising methods for salt-and-pepper noise, such as median filter or AT2F, work well on this noise model. We conducted the experiment using various noise levels from 5% to 50%. For the salt-and-pepper noise, our proposed method performed better than state-of-the-art methods because of its ability to overcome the problem of brightness shifting, as shown in Figure 5 (third row). Please note that other blind denoising methods (N2V, N2S, and S2S) performed poorly on this noise model. Furthermore, our proposed method outperformed AT2F when d≥15 on Set14, as shown in Figure 5. Similar to speckle noise, blind denoising methods (i.e., N2V, N2S, and S2S) failed to restore the image contrast of the clean image but our method successfully preserved the contrast and brightness of the original image. Moreover, the proposed method shows better performance as the noise level increases. Note also that the results of AT2F look much blurrier than those of the proposed method.

### 4.2. Denoising Results on Fusion Noise (Unknown Noise Statistics)

In this section, we compare the performance of denoising methods when the prior knowledge of noise statistics is not available. For this, we generated the fusion noise, which is a mixture of different noise models. We combined three known noise models, AWGN, speckle noise, and salt-and-pepper noise, with $\sigma_{g}$, $\sigma_{s}$, and *d* to simulate this fusion noise, which is formally defined as follows:

(16) $$\begin{array}{c} y=f_{spn}\left(\left(x+n_{g}\right)+n_{s}*\left(x+n_{g}\right),d\right) \end{array}$$

(17) $$\begin{array}{c} n_{g}∼N\left(0,\;\sigma_{g}^{2}\right)\;,n_{s}∼U\left(0,\;\sigma_{s}^{2}\right)\;. \end{array}$$

To compare the results on various noise levels, we selected $\sigma_{g}\in \left\{25,50\right\}$, $\sigma_{s}\in \left\{5,25\right\}$, and d∈{5,25}.

We compare our proposed method with three well-known blind denoisers (N2V, N2S, and S2S), along with N2C (supervised denoiser) as a baseline. For highly corrupted images such as fusion noise, the image prior knowledge related to gradient can improve the denoising performance to restore structured artifact that could be restored by total variation minimization. The structured artifact was also reported in N2V, and remains as a limitation of the proposed method in this paper. Hence, to resolve this limitation, we employed a TV regularization term as shown below:

(18) $$\begin{array}{c} L\left(f,x\right)=E_{j}\left[w_{j}{\left(f{\left(x\right)}_{j}-x_{j}\right)}^{2}\right]+{\alpha ||f\left(x\right)||}_{TV} \end{array}$$

**Figure 6:**
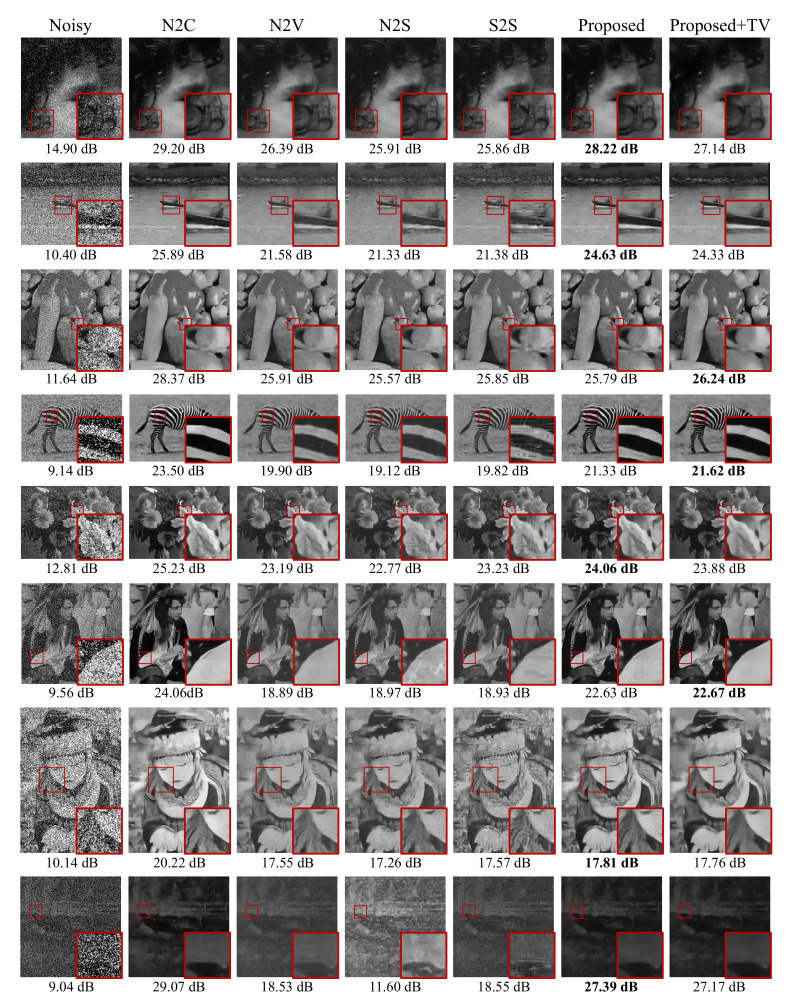
Qualitative performance comparison of various denoising methods on fusion noise. Top to bottom ($\sigma_{g}$, $\sigma_{s}$, d): (25,5,5), (25,5,25), (25,25,5), (25,25,25), (50, 5, 5), (50, 5, 25), (50, 25, 5), (50, 25, 25), respectively. The best PSNR is highlighted in bold, except N2C.

We empirically found the value ($1\times 10^{-7}$) of alpha that the scale of total variation can reach similar to the scale of adaptive loss. As shown in Figure 6, all other blind denoising methods inaccurately reconstructed the black color to brighter gray color. N2S and S2S also suffered from structural artifacts as well as incorrect brightness (Figure 6 last row). We observed that our model predicted the clean image more accurately while preserving the image contrast and details well as compared with N2S, N2V, and S2S. Furthermore, the TV added version, called N2K+TV, effectively removed noise while preserving sharp edges.

Table 1 and Table 2 summarize the results for various noise levels and denoising methods; our proposed method with total variation (N2K+TV) achieved the highest PSNR compared with the state-of-the-art blind denoising methods. It is clearly shown that the TV regularization effectively improves SSIM, especially for the higher noise levels. We also observed that the performance gap between our method and the others becomes larger as the noise level increases. In summary, we conclude that the proposed method with total variation regularization overcomes the problems caused by the fusion noise that affects most other denoising methods.

### 4.3. Ablation Study

In this section, we empirically show the difference in the performance of ADSS loss against the general self-supervision loss. In this experiment, we used the same network structure for all test cases; however, the network was trained using different loss functions to see how they affected the performance. The baseline model was trained using the general self-supervision $L_{2}$ loss Equation (1).

Table 3 shows the results of the previously introduced models when tested on the BSD68 dataset. It can be seen that the ADSS loss, which suppresses training from outliers, outperforms the general self-supervision loss at various levels of fusion noise except the case of $\sigma_{g}$ = 50, $\sigma_{s}$ = 5, and *d* = 5. In addition, ADSS+TV achieved higher PSNR and SSIM than the baseline and ADSS alone. As the general self-supervision loss considers all pixels to be training data, it is more sensitive to highly corrupted noisy pixels. The study result also confirms that the performance gap between baseline and ADSS is bigger for higher noise levels (d=25). As shown in the unknown noise statistics experiment, we observed that the TV loss helped to increase PSNR and SSIM in highly corrupted images. In this ablation study, we demonstrated that the ADSS loss outperformed the general self-supervision loss. We also observed that the TV regularization was highly effective at further improving the image quality. Additionally, TV can reduce the artifact from structured noise, which is the weakness of the proposed method.

### 4.4. Analysis for ADSS

The basic concept of the ADSS loss is to reduce the unnecessary training from noisy pixels which have a large mean squared error. We now show simple examples highly related to decrease of redundant training. The following examples provide more insights into the properties of the proposed ADSS loss.

**Figure 7:**
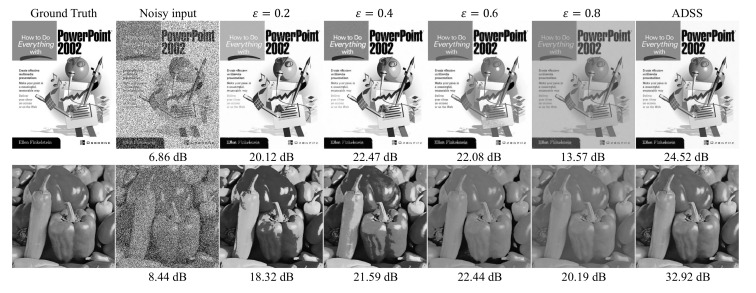
Effect of the loss clipping for various threshold values on salt-and-pepper noise (d = 50).

**Figure 8:**
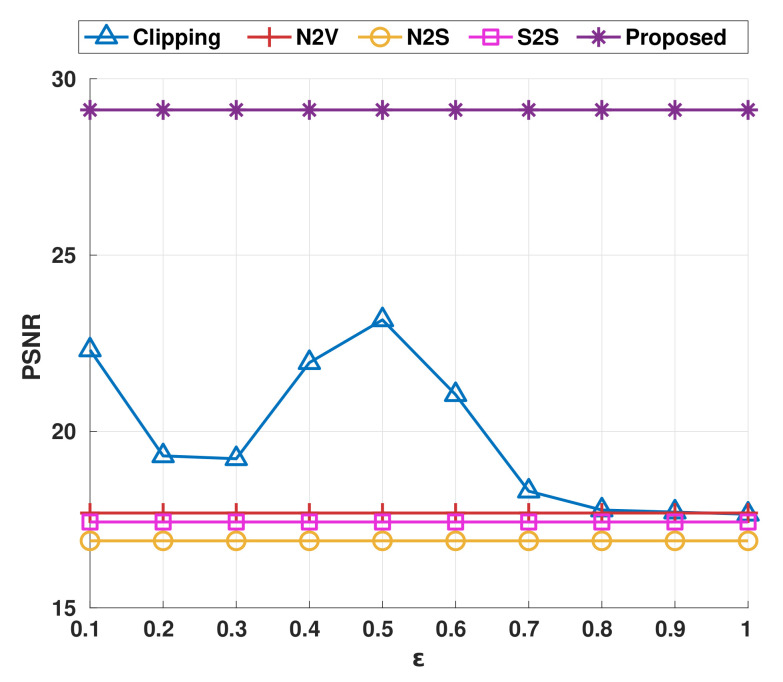
Average PSNR graph of loss clipping on Set 14 in terms of ε.

First, we should assume that the large gap between $x_{j}$ and $f{\left(x\right)}_{j}$ for an arbitrary given *j* implies learning from $x_{j}$ disturb the denoising performance of the function *f* (i.e., *f* is close to an ideal denoiser). Then, we simply reduce the loss forcibly by clipping of the loss as

(19) $$\begin{array}{c} L_{clip}\left(f,x\right)=E_{j}\left[clip\left({\left(f{\left(x\right)}_{j}-x_{j}\right)}^{2},0,\varepsilon \right)\right],\varepsilon >0, \end{array}$$

over the clip function that limits the loss exceeds a threshold ε. We conducted an additional experiment to analyze ADSS loss indirectly through the clipping method under the same conditions of salt-and-pepper noise experiments as Section 4.1.3. On the top row of Figure 7, trained denoisers by adjusted loss with ε∈{0.2,0.4,0.6} successfully recovered white background, unlike the results of state-of-the-art blind denoising methods reported in Section 4.1.3. Additionally, the loss clipping led to better PSNR compared with state-of-the-art blind denoising approaches, as shown in Figure 8. We discovered that the reduction of the loss from the pixels with a large gap between $x_{j}$ and $f{\left(x\right)}_{j}$ boosts the performance on salt-and-pepper noise. Unfortunately, the clipping method requires proper value of ε to obtain correct restoration result. Moreover, ε should be found for each pixel locally instead of global thresholding. On the bottom row of Figure 7, we discovered an over emphasized contrast on the pepper surface for significant loss clipping (where ε=0.2 and 0.4). In summary, we empirically showed that the reduction of the loss can prevent unnecessary learning from unpredictable noise (e.g., salt-and-pepper noise and fusion noise) in the general self-supervision loss. Moreover, the ADSS loss can successfully adjust the size of self-supervision loss adaptively and automatically for each pixel.

## 5. Conclusions

We introduced a novel unsupervised denoising method based on the dilated convolutional J-invariant network, allowing for efficient kernel-based training without the masking scheme. The absence of preprocessing further pushes the performance in terms of training efficiency. We also proposed an adaptive self-supervision loss that is highly effective in preserving overall brightness and structures in the image, even with the extremely high noise level and even if the zero-mean assumption and prior knowledge of noise statistics are not present. Using simulations of known and unknown noise statistics, we showed that the proposed method leads to better denoising quality than other state-of-the-art methods of blind denoising.

We believe the proposed work will be useful in improving highly corrupted noisy images where noise statistics are not readily available. Extending the proposed architecture to general image enhancement problems, such as blind image super-resolution, is another interesting future work. As a limitation, we found no significant performance improvement on AWGN experiment and low-level noisy images such as DND [38] dataset. In the future, we plan to develop more improved ADSS loss, such as the exponential form to outperform the state-of-the-art blind denoising methods on AWGN and real noisy dataset. Furthermore, we plan to explore applications of our method, especially in the biomedical imaging domain.

## Figures and Tables

**Table 1:** Performance of baselines, the proposed method and the proposed method+TV on the BSD68 test set. Boldface denotes the best among all except N2C.

Noise Level	$\sigma_{g}$ = 25, $\sigma_{s}$ = 5, *d* = 5		$\sigma_{g}$ = 25, $\sigma_{s}$ = 5, *d* = 25		$\sigma_{g}$ = 25, $\sigma_{s}$ = 25, *d* = 5		$\sigma_{g}$ = 25, $\sigma_{s}$ = 25, *d* = 25	
Method\Metric	PSNR	SSIM	PSNR	SSIM	PSNR	SSIM	PSNR	SSIM
N2C	26.99	0.7588	26.24	0.7303	24.68	0.6673	23.96	0.6353
N2V	24.61	0.6817	20.96	0.5908	21.88	0.5940	19.29	0.5111
N2S	24.42	0.6789	21.16	0.5879	21.49	0.5727	19.03	0.4896
N2K (ours)	**25.28**	**0.6892**	**24.52**	0.6435	22.42	0.5580	21.46	0.4869
N2K+TV (ours)	25.13	0.6853	24.42	**0.6513**	**22.61**	**0.6043**	**21.86**	**0.5673**
**Noise Level**	$\sigma_{g}$ **= 50,** $\sigma_{s}$ **= 5,** d **= 5**		$\sigma_{g}$ **= 50,** $\sigma_{s}$ **= 5,** d **= 25**		$\sigma_{g}$ **= 50,** $\sigma_{s}$ **= 25,** d **= 5**		$\sigma_{g}$ **= 50,** $\sigma_{s}$ **= 25,** d **= 25**	
Method\Metric	PSNR	SSIM	PSNR	SSIM	PSNR	SSIM	PSNR	SSIM
N2C	25.21	0.6782	24.38	0.6391	23.85	0.6225	22.96	0.5810
N2V	22.64	0.5930	19.83	0.5337	20.57	0.5444	18.48	0.4794
N2S	22.00	0.5746	19.71	0.4999	19.95	0.5141	18.41	0.4404
N2K (ours)	**23.40**	0.6038	22.55	0.5471	20.49	0.5063	19.73	0.4321
N2K+TV (ours)	**23.40**	**0.6149**	**22.67**	**0.5786**	**20.59**	**0.5635**	**19.82**	**0.5195**

**Table 2:** Performance of baselines, the proposed method and the proposed method+TV on the Set14 test set. Boldface denotes the best among all except N2C.

Noise Level	$\sigma_{g}$ = 25, $\sigma_{s}$ = 5, *d* = 5		$\sigma_{g}$ = 25, $\sigma_{s}$ = 5, *d* = 25		$\sigma_{g}$ = 25, $\sigma_{s}$ = 25, *d* = 5		$\sigma_{g}$ = 25, $\sigma_{s}$ = 25, *d* = 25	
Method\Metric	PSNR	SSIM	PSNR	SSIM	PSNR	SSIM	PSNR	SSIM
N2C	28.06	0.7749	27.23	0.7476	25.61	0.6918	24.79	0.6615
N2V	25.51	0.7074	20.93	0.6089	22.59	0.6199	19.32	0.5335
N2S	24.06	0.6683	20.40	0.5805	21.34	0.5797	18.68	0.4971
S2S	25.72	**0.7256**	20.88	0.5951	22.58	0.6252	19.27	0.5149
N2K (ours)	**26.42**	0.7169	**25.46**	0.6674	23.25	0.5782	22.19	0.4992
N2K+TV (ours)	26.26	0.7163	25.33	**0.6791**	**23.52**	**0.6372**	**22.67**	**0.5966**
**Noise Level**	$\sigma_{g}$ **= 50,** $\sigma_{s}$ **= 5,** d **= 5**		$\sigma_{g}$ **= 50,** $\sigma_{s}$ **= 5,** d **= 25**		$\sigma_{g}$ **= 50,** $\sigma_{s}$ **= 25,** d **= 5**		$\sigma_{g}$ **= 50,** $\sigma_{s}$ **= 25,** d **= 25**	
Method\Metric	PSNR	SSIM	PSNR	SSIM	PSNR	SSIM	PSNR	SSIM
N2C	26.16	0.7029	25.19	0.6631	24.66	0.6497	23.62	0.6082
N2V	23.01	0.6192	19.67	0.5572	20.88	0.5699	18.31	0.5005
N2S	21.65	0.5714	19.07	0.4994	19.26	0.5103	17.76	0.4438
S2S	23.31	0.6441	19.64	0.5361	21.04	0.5725	18.35	0.4748
N2K (ours)	**24.24**	0.6305	23.22	0.5708	21.20	0.5248	20.38	0.4438
N2K+TV (ours)	**24.24**	**0.6471**	**23.35**	**0.6076**	**21.31**	**0.5944**	**20.49**	**0.5452**

**Table 3:** Comparison of ADSS and general self-supervision loss. Average PSNR and SSIM for fusion noise on BSD68 validation set. The baseline uses only the structure of the proposed method with general self-supervision $L_{2}$ loss. Boldface denotes the best performance among Baseline, ADSS, and ADSS +TV.

Model	Baseline		ADSS		ADSS + TV	
Noise Level\Metric	PSNR	SSIM	PSNR	SSIM	PSNR	SSIM
$\sigma_{g}$ = 25, $\sigma_{s}$ = 5, *d* = 5	24.54	0.6761	**25.28**	**0.6892**	25.13	0.6853
$\sigma_{g}$ = 25, $\sigma_{s}$ = 5, *d* = 25	20.93	0.5577	**24.52**	0.6435	24.42	**0.6513**
$\sigma_{g}$ = 25, $\sigma_{s}$ = 25, *d* = 5	21.66	0.5679	**22.42**	0.5580	21.61	**0.6043**
$\sigma_{g}$ = 25, $\sigma_{s}$ = 25, *d* = 25	19.22	0.4850	21.46	0.4869	**21.86**	**0.5673**
$\sigma_{g}$ = 50, $\sigma_{s}$ = 5, *d* = 5	22.54	0.5872	**23.40**	0.6038	**23.40**	**0.6149**
$\sigma_{g}$ = 50, $\sigma_{s}$ = 5, *d* = 25	19.71	0.5162	22.55	0.5471	**22.67**	**0.5786**
$\sigma_{g}$ = 50, $\sigma_{s}$ = 25, *d* = 5	**20.59**	0.5390	20.49	0.5063	**20.59**	**0.5635**
$\sigma_{g}$ = 50, $\sigma_{s}$ = 25, *d* = 25	19.22	0.4850	21.46	0.4869	**21.86**	**0.5673**

## Data Availability

The data are from the public datasets. BSD400 and BSD68 are available in https://www2.eecs.berkeley.edu/Research/Projects/CS/vision/bsds/ (accessed on 26 May 2022). Set14 is available in https://github.com/jbhuang0604/SelfExSR (accessed on 26 May 2022).
